# “*Altiarchaeales*”: Uncultivated Archaea from the Subsurface

**DOI:** 10.3390/life5021381

**Published:** 2015-05-12

**Authors:** Alexander J. Probst, Christine Moissl-Eichinger

**Affiliations:** 1 Department of Earth and Planetary Science, University of California, Berkeley, 307 McCone Hall, Berkeley, CA 94720, USA; E-Mail: alexander.j.probst@gmail.com; 2 Interactive Microbiome Research, Section of Infectious Diseases and Tropical Medicine, Department of Internal Medicine, Medical University Graz, Graz 8036, Austria; 3 BioTechMed, Krenngasse 37, Graz 8010, Austria

**Keywords:** Archaea, subsurface, uncultivated, hami, sulfidic springs

## Abstract

Due to the limited cultivability of the vast majority of microorganisms, researchers have applied environmental genomics and other state-of-the-art technologies to gain insights into the biology of uncultivated Archaea and bacteria in their natural biotope. In this review, we summarize the scientific findings on a recently proposed order-level lineage of uncultivated Archaea called Altiarchaeales, which includes “*Candidatus* Altiarchaeum hamiconexum” as the most well-described representative. *Ca*. A. hamiconexum possesses a complex biology: thriving strictly anaerobically, this microorganism is capable of forming highly-pure biofilms, connecting the cells by extraordinary cell surface appendages (the “hami”) and has other highly unusual traits, such as a double-membrane-based cell wall. Indicated by genomic information from different biotopes, the Altiarchaeales seem to proliferate in deep, anoxic groundwater of Earth’s crust bearing a potentially very important function: carbon fixation. Although their net carbon fixation rate has not yet been determined, they appear as highly abundant organisms in their biotopes and may thus represent an important primary producer in the subsurface. In sum, the research over more than a decade on *Ca.* A. hamiconexum has revealed many interesting features of its lifestyle, its genomic information, metabolism and ultrastructure, making this archaeon one of the best-studied uncultivated Archaea in the literature.

## 1. The Discovery of the Altiarchaeales

*Candidatus* Altiarchaeum hamiconexum (formerly known as SM1 Euryarchaeon) was discovered almost 15 years ago in a swamp-area in Germany (Sippenauer Moor, near Regensburg [1]). Forming the so-called string-of-pearls community together with filamentous bacteria, it was found to thrive in the surface waters of sulfidic, cold springs. These string-of-pearls communities were eye-catching structures, attached on one end to solid surfaces and floating in the water streamlet. The inner part of the pearls was formed by a microcolony of the Archaea, which was encased by a dense network of filamentous *Thiothrix* sp., a sulfide-oxidizing microorganism [2]. Since both partners seemed to specifically seek each other, syntrophy (based on an internal nutrient exchange) or oxygen removal by the bacterial partner for the benefit of the Archaea was proposed [2].

Fairly soon after this discovery, another biotope of *Ca.* A. hamiconexum and of the string-of-pearls communities was found. In contrast to the first biotope, this cold, sulfidic groundwater emanated from a 36.5-m deep, drilled hole (Mühlbacher Schwefelquelle; [3,4]). This aquifer delivers ~5400 L of sulfidic freshwater per hour and transports thereby subsurface microbes from the deep to the surface, thus allowing indirect access to the subsurface. By providing attachment devices to the water flow at about a 1-m depth, biofilm pieces were filtered from the upwelling water. These biofilms consisted almost exclusively of *Ca.* A. hamiconexum (95%) and, to a very low amount, of diverse, mainly sulfate-reducing bacteria (5%; [5,6]). Until that date, the capability of Archaea to form highly-pure biofilms in natural environments had not been described. Additionally, a natural predominance of one species of Archaea in a habitatwas indicated [5].

The discovery of this biofilm enabled research on these subsurface Archaea, even without being able to grow them in the laboratory. Until today, these Archaea cannot be stably grown in culture, a fact that was overcome by sampling high amounts of their biomass from the natural environment. The high biomass enabled an analysis of the biology of these Archaea without introducing bias from artificial growth conditions *in vitro*.

In a very recent publication, we proposed the name *Ca.* A. hamiconexum for the former SM1 Euryarchaeon. Its phylogenetic clade was named Altiarchaeales, since it formed a separate order-level branch next to the Methanococcales [7]. Until today, three major representatives of the Altiarchaeales have been mentioned in the literature, each of them with a reconstructed genome. The best-described organism is *Ca.* A. hamiconexum (strain SM1-MSI, from the above-mentioned sites), with a close relative sampled from a U.S. American spring named *Ca.* A. hamiconexum strain SM1-CG. Another Altiarchaeales representative was detected as a minor component of the subsurface biofilm of *Ca.* A. hamiconexum and named IM-C4 [7].

In the following chapters, we will summarize the major findings of the last 15 years and add some additional information about the ecology and distribution of these unusual subsurface Archaea.

## 2. The Life Styles of *Ca.* Altiarchaeum Hamiconexum

It all starts with an unknown ecological niche somewhere in the terrestrial subsurface. At least 20, 30 meters in depth, *Ca.* A. hamiconexum cells are metabolically active and reproduce most probably with high efficiency, thus forming a large amount of biomass as a biofilm [7]. Within this biofilm, other organisms besides *Ca*. A. hamiconexum are successfully outcompeted or even actively suppressed by unknown mechanisms of *Ca.* A. hamiconexum. From time to time, randomly or purposely, biofilm pieces are released, which are then transported to the surface in the water flow. There, they seek companionship with filamentous sulfide-oxidizing bacteria, which cover the surface of the microcolony entirely, forming the string-of-pearls community [2,4]. In these communities, the Archaea are capable of proliferating for a few days, after which the assemblages lose their structure and the microbes disperse into the streamlet water [4,8].

After one and a half decades of research on *Ca.* A. hamiconexum, there remain many unanswered questions. For instance, it is unexplored why and how the Archaea keep control over the minor bacterial fraction in the biofilm and if there exists any type of interaction between these organisms, which may be the basis for symbiosis/syntrophy rather than competition. Regarding the string-of-pearls community at the surface, the triggers for the attachment of sulfide-oxidizing bacteria to the biofilm pieces and potential, subsequent changes in the metabolic functions of both partners remain uninvestigated.

Nevertheless and to the best of our knowledge, this is the first archaeal organism showing a change in its lifestyle in a natural biotope. This change in lifestyle has been very well documented across sampling sites [4] and could be used as a model system to investigate anaerobic Archaea experiencing drastic geochemical changes in their natural environment.

## 3. Biofilm and Cell Structure

**Figure 1:**
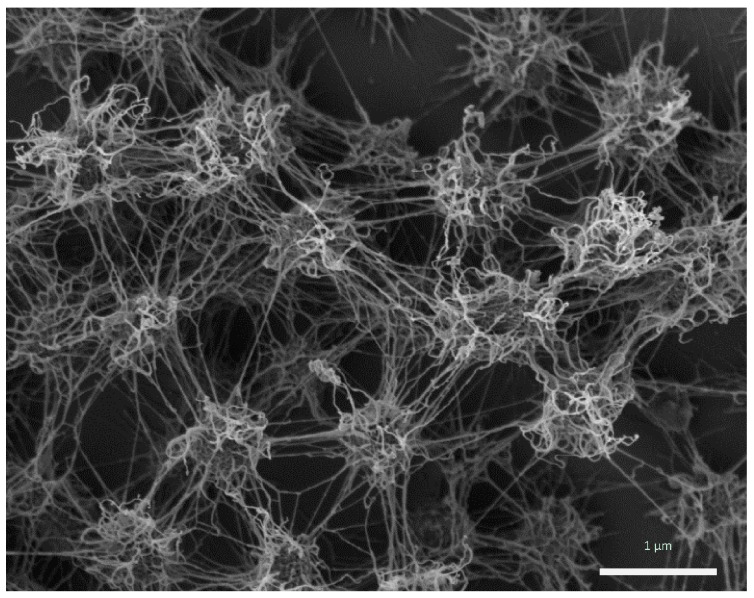
*Ca.* A. hamiconexum cells within their biofilm (Sippenauer Moor biofilm; scanning electron micrograph). Cells appear fluffy due to their extracellular polymeric matrix and cell-surface appendages (“hami”).

The porous, but rigid and well-structured biofilm of *Ca.* Altiarchaeum hamiconexum (Figure 1) was formed by coccoid cells (diameter: 0.6 μm), which were arranged in regular, three-dimensional patterns with distances of approximately 2 μm [4,7,9].

**Figure 2:**
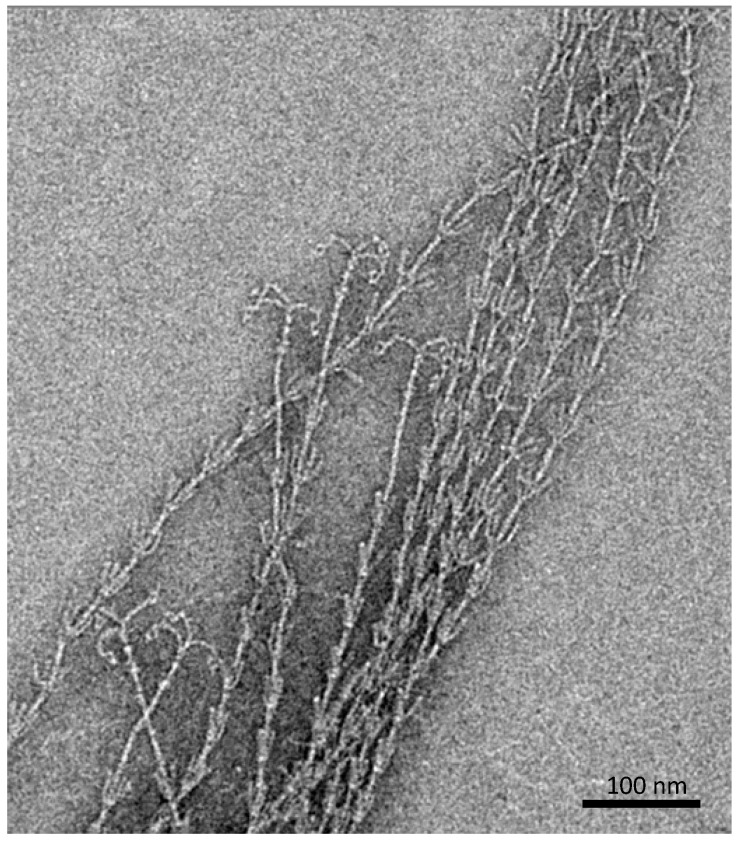
Barbed-wire like cell surface appendages of *Ca*. A. hamiconexum with grappling hooks (hami; Sippenauer Moor biofilm; electron micrograph).

Besides an extracellular, protein- and carbohydrate-rich matrix [6], which is attached to the cell surface and reveals a thickness of about 600 nm, proteinaceous, filamentous cell surface appendages (called “hami”, singular “hamus”; Latin for hook; Figure 2) mediate the strong connections between cells, but also between cells and biotic or abiotic surfaces [10].

The hami are up to 5 μm in length and carry a barbed grappling hook at their distal end. The entire filament is about 7 nm in diameter and reveals prickles emanating from the major filament, which resembles a barbed wire structure. Overall, such surface structures have never been observed for any other life form. The grappling hooks represent the smallest biologically-formed hooks ever discovered and thus possess great potential for application in nano-biotechnology [11]. Noteworthy, the hami appear to be composed of only one major protein species, which does not show any homologies to known microbial proteins involved in cell motility or adhesion. However, the protein sequence and structure does reveal similarities to S-layer proteins of Archaea, posing the question of whether hami have evolved from these proteins or *vice versa* [12]. Since the hami are anchored in a double-membrane (a quite unusual cell wall type for Archaea [13]), one could even speculate about a parallel loss of an S-layer sheath and the development of a second membrane to ensure cell integrity.

Although the hami are probably the best-characterized cell surface appendages of an uncultivated organism, many questions remain unanswered, particularly those that necessitate cultivation and establishment of a genetic system of the organism. First of all, it is unclear, if and what other types of proteins are involved in the hami-anchorage and formation and how hami formation is regulated within the cell. In addition, any components of the outer membrane remain unknown, which holds true for most of the other, rare, double-membraned Archaea, such as *Methanomassiliicoccus luminyensis* [14].

To date, also a possible function of the hami besides attachment remains elusive. Most microbes have developed pili or flagella to strongly attach to different surfaces [15], and such structures seem to provide enough adhesion power due to van-der-Waals forces prevailing at such small dimensions. The hami, however, are highly structured and bear a grappling hook at the end, whose specific function is unknown so far. The structural analyses of the protein have indicated the possible capability towards conformational change [12], but it remains to be analyzed whether the hami are involved in signal transduction or may even mediate electron transfer, as discussed recently [7]. 

Since biofilm formation usually requires some communication between cells (quorum sensing) [16] and the porous biofilm of *Ca.* A. hamiconexum thriving in groundwater with high flow rates may not allow communication via small molecules between cells, we hypothesize that the hami could fulfill such a communication role, *i.e.*, via tractive forces. The hooks may ensure anchorage to another cell or another (a)biotic surface. We speculate that traction applied to the hooks by cells in an environment with high pressure and flow rate could initiate an intra-cellular signal cascade. Such a signal cascade could enable cells to form biofilms via cell division and initiate other factors, like production of extracellular polymeric substances (EPS).

*Ca.* A. hamiconexum strains SM1-MSI and SM1-SM are the only Altiarchaeales that have been visualized thus far, with the exception of IM-C4. The latter is present in the biofilm as a very minor constituent and was recently detected via fluorescence *in situ* hybridization [7]. Similar to SM1-MSI and SM1-SM, these cells were very small (<1 μm) and revealed a coccoid shape.

## 4. The Phylogeny and Relatives

*Ca.* Altiarchaeum hamiconexum had early been identified to belong to the Euryarchaeota phylum based on 16S rRNA gene sequence analysis [1]. However, finding a stable position of the 16S rRNA gene within this phylum when applying treeing methods appeared to be problematic. Due to its long branch arising from genomic undersampling of close relatives, the sequence tended to cluster with other fast-evolving Archaea, such as Nanoarchaeota, but did not reveal a stable branch in the tree [1]. Using a phylogenomics approach based on concatenated ribosomal protein sequences and state-of-the-art treeing methods, we were able to position *Ca.* Altiarchaeum hamiconexum close to the methanogen origin, most probably as a sister group to Methanococcales [7]. When applying this phylogenomics approach, genomic data of Altiarchaeales IM-C4 enabled us to stabilize the branch of this order [7].

The distribution of the order Altiarchaeales across several environments was captured based on available 16S rRNA gene sequences, which formed a distinct cluster [7]. On the basis of this analysis, the Altiarchaeales group appeared to be composed of widespread and multiple lineages. The closest relative of *Ca.* Altiarchaeum hamiconexum was found to be SM1-CG (~97% identity of the 16S rRNA gene), which had been detected in filtration samples from a CO_2_-driven, cold geyser in the U.S. (Crystal Geyser, [7]).

Other, more distantly related 16S rRNA gene sequences were obtained from hot springs in the U.S. (13% difference in the 16S rRNA gene) and Bulgaria (11% difference). The former sequences were obtained from groundwater emanating from a drilled, >200-m deep hole (Lidy Hot Springs, Idaho; [17]). The system was described to be more or less free of organic compounds, but rich in hydrogen. The water itself had a temperature of about 58.8 °C and was anoxic. Microbial communities were obtained by filtering the water, and subsequent quantitative PCR revealed the predominance of Archaea (95%). The authors, however, placed the obtained 16S rRNA gene fragments close to the methanogens, proposed a methanogenic, hydrogen-consuming lifestyle for the corresponding microorganisms and discussed analogies to possible extraterrestrial habitats [17]. The latter sequences were derived from the Rupi Basin, Bulgaria (spring RB; [18]): its groundwater revealed a temperature of about 79 °C, being fed by subsurface hydrothermal fluids. The water was found to be rich in sulfates (indicative of sulfur-oxidation activity) and elemental sulfur, as well as higher concentrations of nitrate and nitrite.

In sum, the entire Altiarchaeales clade seems to be composed of Archaea thriving in anoxic freshwater and deep-sea environments, including plant reservoirs and sediments [7].

## 5. The Distribution of *Ca.* Altiarchaeum Hamiconexum in Southern German Springs

In order to better understand the distribution of Altiarchaeales in natural habitats and preferences with respect to chemical and physical parameters, we performed a screening of 15 different springs, all located in southern Germany (Table 1; see also [7]). The springs were chosen to cover the broadest diversity with respect to geographical setting and physicochemical parameters. For instance, eight springs did not reveal the presence of sulfide (below the detection limit), and two springs were characterized by the presence of high amounts of ferric iron precipitating near the spring outlet (Schwärz (AE), Frauenhäusl (FH); Table 1). All springs were geologically distributed in the area of Regensburg (area diameter: 30 km); some were located north (AE, Faulwies (FW), Aschach (AS)) and the others south of the Danube. Thus, the selected springs had different geological and geochemical settings.

In brief, 5.0 L of spring water were taken and subjected to filtration. Filters (Milliflex Filtration Funnels, 0.45 µm) were then used for DNA extraction [19], and qPCR was performed as previously described [6]. We focused on the detection of the genus Altiarchaeum using 16S rRNA gene-targeting primers for the closest phylogenetic group around *Ca.* Altiarchaeum hamiconexum (forward: 344F, [20]; SMgroup_443r: 5'-CGCAGTGCTTCTTACACAC-3'). The results are given in Table 1.

Considering all springs that revealed 16S rRNA gene sequences of the SM1 cluster in qPCR analysis, a correlation of oxygen concentration and percentage of qPCR values across Archaea (Table 1) revealed a significant negative linear correlation (Pearson correlation, *p*-value < 0.05). In other words, the lower the oxygen concentration, the greater the abundance of the *Ca.* Altiarchaeum hamiconexum-group within the Archaea in these springs. Consequently, it seems that these organisms are either outcompeted under oxygen-rich conditions or can be seen as strictly anaerobic microorganisms, whose cellular abundance declines when oxygen is present. This analysis supports the observation from previous studies [4,7] and is backed up by diversity and genomic analyses (see below).

**Figure 3:**
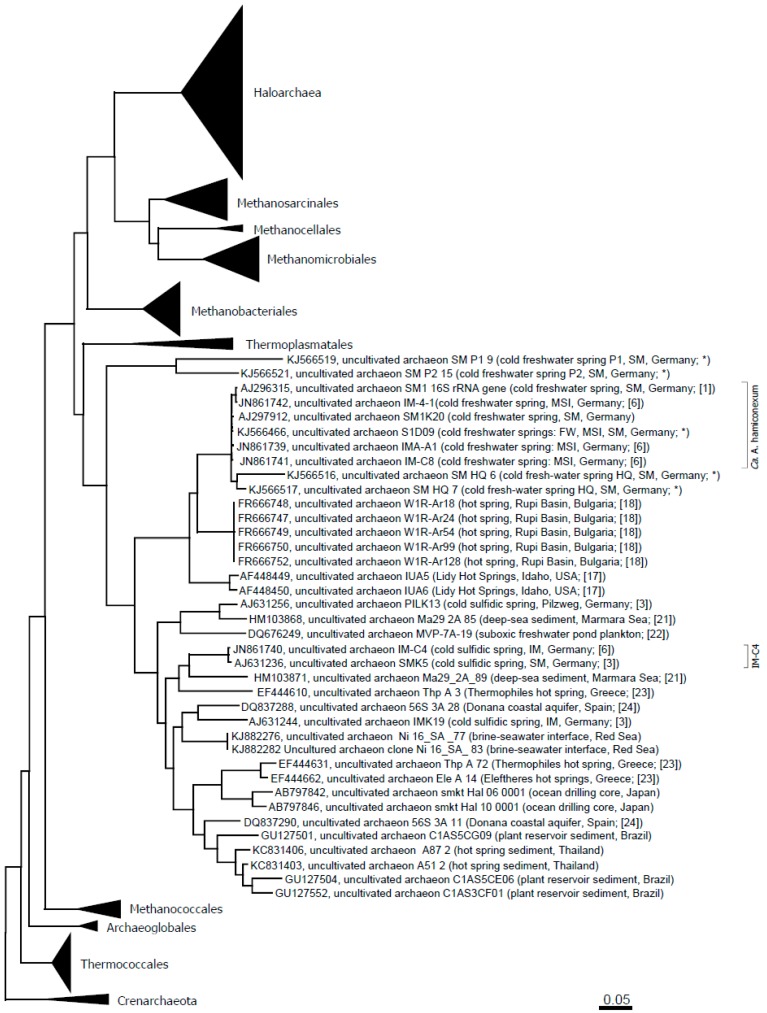
Altiarchaeales within the Euryarchaeota tree and in context to published sequences from additional, diverse environments [21,22,23,24]. The evolutionary history was inferred using the maximum likelihood method. The tree is drawn to scale, with branch lengths measured in the number of substitutions per site. The analysis involved 386 nucleotide sequences. All positions with less than 95% site coverage were eliminated. That is, fewer than 5% alignment gaps, missing data and ambiguous bases were allowed at any position. There were a total of 968 positions in the final dataset. Evolutionary analyses were conducted in MEGA6 [25]. Abbreviations: SM: Sippenauer Moor; MSI: Muehlbacher Schwefelquelle Isling; FW: Faulwies. * This study.

For selected spring waters, the archaeal diversity was assessed via phylum-targeting 16S rRNA gene sequencing (Table 2). Archaea were detectable in all of the different spring waters, with Euryarchaeota being the most prevalent (Table 2). In particular, methanogenic Archaea were found in the majority of the springs. Besides Euryarchaeota, also Crenarchaeota and Thaumarchaeota were found. *Ca.* genus Altiarchaeum was detected in almost all springs from the Sippenauer Moor (the marsh environment where these Archaea were discovered first) and also in Mühlbacher Schwefelquelle Isling (MSI) and FW. Figure 3 depicts an updated phylogenetic tree of the Altiarchaeales.

**Table 1:** An overview of all springs, their physical and chemical parameters, as well as the detection of Archaea and the Sippenauer Moor (SM) group. n.d.: not determined. bdl: below the detection limit. * Selected for qualitative analyses using on 16S rRNA gene cloning. ^a^ Calculation was inferred from the many biofilm pieces washed up from the subsurface, which were sometimes not caught by simple water collection.

Sampling Area	Exact Location	Presence of Precipitates and Bio-Material	Abbreviation for spring location	pH	Temperature in °C	Oxygen (% Air Saturation)	S_2_^−^ in mg/L	Detection of Archaea	Detection of *Altiarchaeum* (% of Archaea)
Sippenauer Moor	Main spring	+ (whitish)	SM_HQ	6.5	11.0	4.3	0.6	+ *	72.49
	Side spring (2)	+ (whitish)	SM_NQ	6.5	12.0	4.3	0.8	+ *	73.84
	Non-sulfidic main spring	-	SM_S	6.5	10.4	57.2	bdl	+ *	3.26
	Road	+ (whitish)	SM_W	6.5	n.d.	n.d.	0.1	+	0.17
	Spring at the parking area (right)	+ (whitish)	SM_P1	6–6.5	11.0	31.2	0.1	+ *	1.35
	Spring at the parking area (left)	-	SM_P2	6.5	10.7	42.6	bdl	+ *	0.31
Burgweinting	Mühlbacher Schwefelquelle Isling	+ (whitish)	MSI	6.5–7	10.5	0	0.6	+ *	75.0 ^a^
	Non-sulfidic spring	-	IM2	7	10.6	44.4	bdl	+ *	0.01
St. Katharinen forest	Schwärz	+ (reddish)	AE	6.5	9.0	100.1	bdl	+ *	0
	Faulwies	-	FW	6.5	9.0	83.1	bdl	+ *	10.76
	Aschach	-	AS	6.5	12.3	78.5	bdl	+	0
Barbing	Sulfur fountain	+ (whitish)	B	6.5–7	16.1	15.0	1.0	+	0.04
Harting	Village fountain	-	H	7.5	11.7	58.2	bdl	+	0.08
Teugn	Forest spring	+ (whitish)	T	7	10.5	3.2	1.3	+	0.02
Kelheim	Frauenhäusl	+ (reddish)	FH	6.5	9.7	33.8	bdl	+ *	0.02

**Table 2:** Detected archaeal signatures in southern German springs (Cren: Crenarchaeota; Eury: Euryarchaeota; Thaum: Thaumarchaeota; Uncl: unclassified archaeal sequences). ^1^ Methanosphaerula; ^2^ Methanobacterium; ^3^ Methanosarcina; ^4^ Methanospirillum; ^5^ Methanosaeta; ^6^ *Ca.* Methanoregula; ^7^ *Ca.* Nitrososphaera; ^8^ *Ca.* Nitrosopumilus.

		Cren	Eury							Thaum		Uncl.
Sampling area	Abbreviation	unclassified lineages	Methanobacteriales	Methanosarcinales	Thermoplasmata	SM1 (*Ca.* A. hamiconexum)	Methanomicrobiales	unclassified	pMC2A384	Nitrososphaerales	Cenarchaeales	
Sippenauer Moor	SM_HQ					+						
	SM_NQ	+		+ ^1^	+	+		+				+
	SM_S					+				+ ^7^	+ ^8^	+
	SM_P1		+ ^2^					+	+	+ ^7^		+
	SM_P2			+ ^3^	+	+		+		+ ^7^	+ ^8^	+
Burgweinting	MSI			+	+	+						+
	IM2				+		+ ^4^	+		+ ^7^		+
St. Katharinen forest	AE	+	+	+, + ^5^			+ ^6^	+				+
	FW					+		+		+ ^7^		+
Kelheim	FH							+				+

Based on these results, FW was discovered as another biotope of *Ca.* Altiarchaeum hamiconexum, which had similar temperatures to the others springs analyzed. In contrast to the SM and MSI biotopes, FW is located north of the Danube River close to Regensburg-Schwaighausen. Originally, FW (Faulwies; in English fouling meadow) used to be a swampland, which is today an area for agricultural use. The moisture of the soil (and upwelling water from the subsurface) is channeled within a drainage system, which is accessible at certain points. At one of these points, the water sample analyzed in this study was taken. Due to this setting, the exact position of the original water outlet is unclear, and a prolonged transportation within the drainage tubing cannot be excluded. This could also explain the comparably high oxygen content of the water and the untraceable sulfide content, although residents living in this area reported a sulfidic odor.

Since all of their subsurface biotopes are currently not accessible for direct sampling and none of the Altiarchaeales could be cultivated to date, questions about the original habitat, their physiology and metabolism remained largely unanswered. Nevertheless, looking at the distribution of Altiarchaeales, it seems obvious that this cluster of microorganisms is composed of anaerobic, most probably chemolithotrophic (deep) subsurface microorganisms.

## 6. Genomics and Metabolism

Until today, two genomes of the *Ca.* genus Altiarchaeum (one from a German site, one from a U.S. site) and one more distantly related genome also within the Altiarchaeales (IM-C4) have been recovered from the environment. While all genomes had an estimated size of approximately 1.5 Mbps, the CG content varied from 32.1% for *Ca.* A. hamiconexum to 48.5% for the IM-C4 genome [7].

The genome of *Ca.* A. hamiconexum (strain SM1-MSI) is the best analyzed of this phylogenetic clade and encodes for a reductive acetyl-CoA pathway (Wood-Ljungdhal pathway) as the keystone of the intracellular carbon metabolism [7]. The basis of the Wood-Ljungdhal pathway in *Ca.* A. hamiconexum is the archaeal version utilizing tetrahydromethanopterin. However, the dehydrogenase and reductase of methylene-tetrahydrofolate that necessitates cofactor F_420_ have been replaced by analogous enzymes working with NAD(P)H known from either methylotrophic bacteria or anaerobic methane oxidizers [7]. Based on lipid measurements that showed a very low delta ^13^C value, it was concluded that *Ca.* A. hamiconexum is an autotrophic organism that uses a modified reductive acetyl-CoA pathway to turn carbon dioxide into organic compounds [7]. The so-generated acetyl-CoA is ultimately available for gluconeogenesis and production of various sugars and serves as the precursor for lipid biosynthesis [7]. The Wood-Ljungdhal pathway is also present in the genome of *Ca.* A. hamiconexum recovered from Crystal Geyser in the USA [7]. Moreover, this pathway is also found in the IM-C4 Altiarchaeales genome, which indicates that it may be a general feature of this clade and confirms the importance of this group regarding autotrophic carbon cycling in the subsurface.

**Figure 4:**
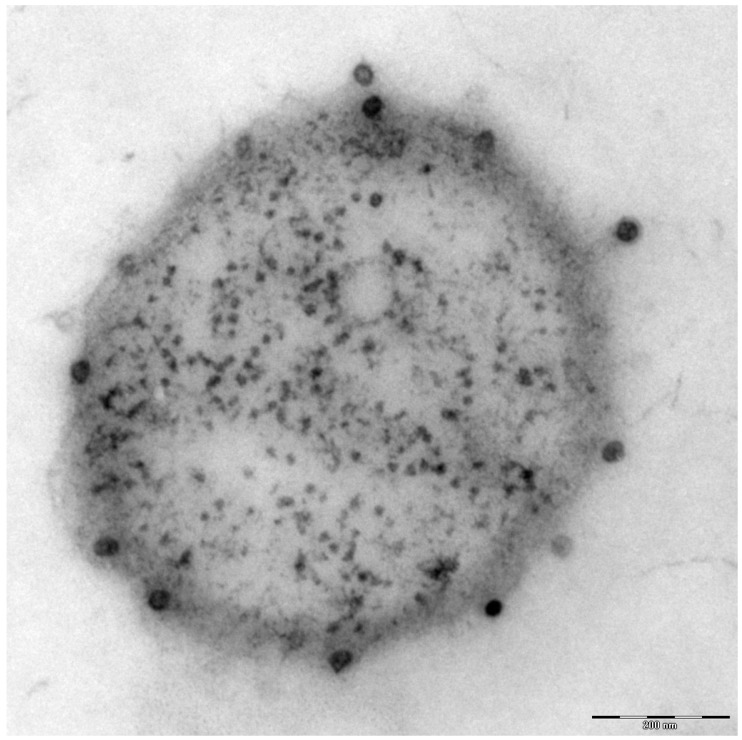
Electron micrograph (thin section): *Ca.* A. hamiconexum with virus-like particles attached to the surface. Bar: 200 nm.

Additionally found in the genome of *Ca.* A. hamiconexum was a clustered, regularly-interspaced short palindromic repeat (CRISPR; [26]) system [7], which is a defense mechanism widespread in bacteria and Archaea against viruses [27]. Although many electron microscopy pictures of thousands of cells have been taken over the course of the past 1.5 decades, only a few micrographs were captured that revealed the potential presence of viruses of *Ca.* A. hamiconexum (Figure 4). To date, it is unknown, whether these viruses play an important role in the biofilms of *Ca.* A. hamiconexum or whether they could even have an indirect, but profound impact on carbon cycling in the subsurface by infecting populations of carbon fixers.

## 7. Cultivation: The Story of Missing Knowledge

Despite numerous attempts, *Ca.* A. hamiconexum can still not be grown in the laboratory as a stable culture. The biofilms are delivered in a putatively healthy status to the spring surface (95% alive, 73% showing FtsZ rings; [5,7]), where they can be retrieved gently by a non-disturbing sampling device [6], capturing cells in a glass bottle under anaerobic conditions. However, even when kept within this closed bottle, the Archaea start dying off fairly soon, and biofilms can be maintained alive up to a max of one week (captured using live/dead staining and fluorescence microscopy). In particular, when nutrients are added (even in extremely low concentrations), bacteria start to overgrow in these closed systems, and even when the bacteria are inhibited by antibiotic mixtures, the Archaea die within a few days. The most extreme loss in viability is observed right at the beginning (within one day after sampling), when more than 99% of all archaeal cells die. The reason for this is unknown; one can speculate about the impact of unintended temperature shifts during transportation, sunlight or other factors.

Due to missing information on the metabolism of *Ca.* A. hamiconexum, we are unable to design culture conditions that mimic its original habitat. We fail to simulate the natural extreme water flow (5400 L per hour) in the laboratory, which could be mandatory for supporting growth by delivery of substrates or removal of metabolic products. In addition, we seem incapable of providing the optimal ranges of required electron donors and acceptors in a steady and sufficient manner, which is particularly important for Archaea, as most of them are specialized for thriving at the knife edge of metabolism and energy gain [28,29].

Although we have recently identified the carbon and nitrogen source used by *Ca.* A. hamiconexum, the question regarding electron donors and acceptors remains unanswered. However, we were able to observe a positive influence of molecular hydrogen in combination with carbon dioxide in qPCR-based incubation studies, although cross-feeding in the community cannot be excluded.

Besides all of these deficiencies in cultivation methodology, the failure of cultivation may be linked to a viable, but non-culturable (VBNC) status of the sampled Archaea rather the unavailability of nutrients. When taken from the upwelling water, the Archaea have undergone rapid shifts in natural conditions (transportation to the subsurface by the water flow), which could have negatively influenced the viability status of the Archaea. Due to experienced changes in chemical and physical conditions, living microbes can enter the VBNC status, in which they are incapable of proliferation [30]. As known from various bacteria, strong triggers are necessary to awaken these microorganisms from VBNC, whereas triggers can differ between even strains [31]. However, such VBNC statuses are completely unknown for Archaea, but could explain the comparably low cultivation success of these microorganisms in general and in particular account for observations made for *Ca.* A. hamiconexum cells under artificial cultivation conditions.

## 8. Conclusions and Outlook

Studying Archaea and bacteria in their natural environment is necessary due to the limited success of cultivating these organisms under laboratory conditions [32]. By studying *Ca.* A. hamiconexum, we have set new standards for the exploration of Archaea in a natural environment. Over the past 15 years, we deciphered different lifestyles of this archaeon [2,4,5]; we discovered novel and unique cell-surface appendages [10], showed the active division of the organism in the environment and deciphered the carbon and nitrogen metabolism using metagenomics and isotopic measurements [7]. Moreover, we studied the metabolism of associated microorganisms to fully grasp potential fluxes happening in the communities of different lifestyles [2,6]. From this work, we concluded that this archaeon could be one of the most dominant organisms in Earth’s anoxic crust and could substantially influence the carbon cycling by fixing carbon dioxide.

With the continuous advancements in technologies used in the field of environmental microbiology, like metagenomics [33] and single cell analyses [34,35], researches will soon be able to study a great diversity of uncultivated microorganisms regarding their biology, including cell structure and metabolism in the actual environment [36], similar to the work summarized in this review for *Ca.* A. hamiconexum.
